# THPLM: a sequence-based deep learning framework for protein stability changes prediction upon point variations using pretrained protein language model

**DOI:** 10.1093/bioinformatics/btad646

**Published:** 2023-10-24

**Authors:** Jianting Gong, Lili Jiang, Yongbing Chen, Yixiang Zhang, Xue Li, Zhiqiang Ma, Zhiguo Fu, Fei He, Pingping Sun, Zilin Ren, Mingyao Tian

**Affiliations:** School of Information Science and Technology, Institution of Computational Biology, Northeast Normal University, Changchun 130117, China; Changchun Veterinary Research Institute, Chinese Academy of Agricultural Sciences, Changchun 130122, China; School of Information Science and Technology, Institution of Computational Biology, Northeast Normal University, Changchun 130117, China; Changchun Veterinary Research Institute, Chinese Academy of Agricultural Sciences, Changchun 130122, China; School of Information Science and Technology, Institution of Computational Biology, Northeast Normal University, Changchun 130117, China; Changchun Veterinary Research Institute, Chinese Academy of Agricultural Sciences, Changchun 130122, China; School of Information Science and Technology, Institution of Computational Biology, Northeast Normal University, Changchun 130117, China; Changchun Veterinary Research Institute, Chinese Academy of Agricultural Sciences, Changchun 130122, China; Changchun Veterinary Research Institute, Chinese Academy of Agricultural Sciences, Changchun 130122, China; School of Information Science and Technology, Institution of Computational Biology, Northeast Normal University, Changchun 130117, China; Department of Computer Science, College of Humanities and Sciences of Northeast Normal University, Changchun 130117, China; School of Information Science and Technology, Institution of Computational Biology, Northeast Normal University, Changchun 130117, China; School of Information Science and Technology, Institution of Computational Biology, Northeast Normal University, Changchun 130117, China; School of Information Science and Technology, Institution of Computational Biology, Northeast Normal University, Changchun 130117, China; School of Information Science and Technology, Institution of Computational Biology, Northeast Normal University, Changchun 130117, China; Changchun Veterinary Research Institute, Chinese Academy of Agricultural Sciences, Changchun 130122, China; Changchun Veterinary Research Institute, Chinese Academy of Agricultural Sciences, Changchun 130122, China

## Abstract

**Motivation:**

Quantitative determination of protein thermodynamic stability is a critical step in protein and drug design. Reliable prediction of protein stability changes caused by point variations contributes to developing-related fields. Over the past decades, dozens of structure-based and sequence-based methods have been proposed, showing good prediction performance. Despite the impressive progress, it is necessary to explore wild-type and variant protein representations to address the problem of how to represent the protein stability change in view of global sequence. With the development of structure prediction using learning-based methods, protein language models (PLMs) have shown accurate and high-quality predictions of protein structure. Because PLM captures the atomic-level structural information, it can help to understand how single-point variations cause functional changes.

**Results:**

Here, we proposed THPLM, a sequence-based deep learning model for stability change prediction using Meta’s ESM-2. With ESM-2 and a simple convolutional neural network, THPLM achieved comparable or even better performance than most methods, including sequence-based and structure-based methods. Furthermore, the experimental results indicate that the PLM’s ability to generate representations of sequence can effectively improve the ability of protein function prediction.

**Availability and implementation:**

The source code of THPLM and the testing data can be accessible through the following links: https://github.com/FPPGroup/THPLM.

## 1 Introduction

Protein thermodynamic stability changes caused by nonsynonymous DNA variations have been shown concerning genetic diseases (Jafri *et al.* 2015, Trezza *et al.* 2017, Hildebrand *et al.* 2020, Pires *et al.* 2020, Xavier *et al.* 2021) and drug resistance (Phelan *et al.* 2016, Hawkey *et al.* 2018, Karmakar *et al.* 2019, Karmakar *et al.* 2020, Portelli *et al.* 2020). At the molecular level, amino acid substitutions in a protein sequence may cause changes in hydrogen bonds, ion-pairs, salt bridges, disulfide bonds, van der Waals interactions, etc., resulting in changes of protein stability (Gromiha and Selvaraj 2004, Kucukkal *et al.* 2015). Moreover, point variations may also cause functional changes. For example, the amino acid residue valine (VAL) at position 483 of the RBM region of the spike glycoprotein changed to alanine (ALA), resulting in changes in secondary structure and relative solvent accessibility (Nguyen *et al.* 2021). Therefore, accurate prediction of changes in protein thermodynamic stability upon single-point variations can promote the study of the biological mechanism of protein structures and association studies between diseases and drugs.

Compared with biological experiments, *in silico* methods are time-saving and cost-saving. The existing methods can be roughly divided into two categories (shown in Supplementary Table S1): (i) structure-based methods, which require protein crystal structure information as input. Some of them, like Rosetta (Chen *et al.* 2020) and FoldX (Guerois *et al.* 2002), calculate protein thermodynamic stability changes (ΔΔ*G*) by simulating the protein folding process. Others, like TML-MP (Cang and Wei 2017), utilize structural information to obtain residue contact networks, atom distances, hydrophobic and hydrophilic areas, etc., to predict ΔΔ*G*; (ii) sequence-based methods, which are mainly learning-based sequence models on the sequence to predict ΔΔ*G* using evolution information or amino acid property, e.g. I-Mutant3.0 (Capriotti *et al.* 2005) and MUpro (Cheng *et al.* 2006).

With the recent progress of pretrained protein language models (PLMs), including ESM-1b (Rives *et al.* 2021), ESM-2 (Lin *et al.* 2023), ProtBert (Elnaggar *et al.* 2022), and UniRep (Alley *et al.* 2019), attention-based architectures, which can effectively extract contextual information from sequences, have been adopted to extract atomic-level structural information from protein sequences and generate representations for specific tasks. In particular, Meier *et al.* (2021) showed that PLMs are capable of modeling the functional effect of sequence variation. Therefore, although existing methods perform well in stability changes prediction, we still want to focus on answering the following questions: (i) Unlike most current sequence-based methods based on manual extract feature engineering, PLM can generate representations for protein sequences, so how can PLM be used to build models that can be used to predict changes in protein stability? (ii) The number and diversity of protein sequences used for training are limited. Can PLM overcome the potential overfitting issues and improve its performance?

In this study, we presented a sequence-based deep-learning framework to predict thermodynamic stability changes upon point variations using PLM, named THPLM. THPLM utilized ESM-2 (Lin *et al.* 2023) to encode 1D protein sequences and a 2-layer CNN to predict ΔΔ*G*. Because of PLM, THPLM generated sequence representations directly from protein sequences, avoiding bias caused by artificial feature engineering. To make an objective evaluation, we re-examined current datasets and built S^sym^148, which removed the duplicates between S^sym^ and the training dataset (S2648). We evaluated THPLM on three independent and unseen testing datasets, and the model showed superior performance compared to other sequence-based methods. Importantly, THPLM achieved state-of-the-art performance of direct Pearson correlation coefficient (PCC) = 0.76, reverse PCC = 0.76, and PCC = 0.86 on the antisymmetric dataset S^sym^148 with experimental structures of wild-type and variant proteins.

## 2 Methods

### 2.1 Datasets

**Figure 1.**
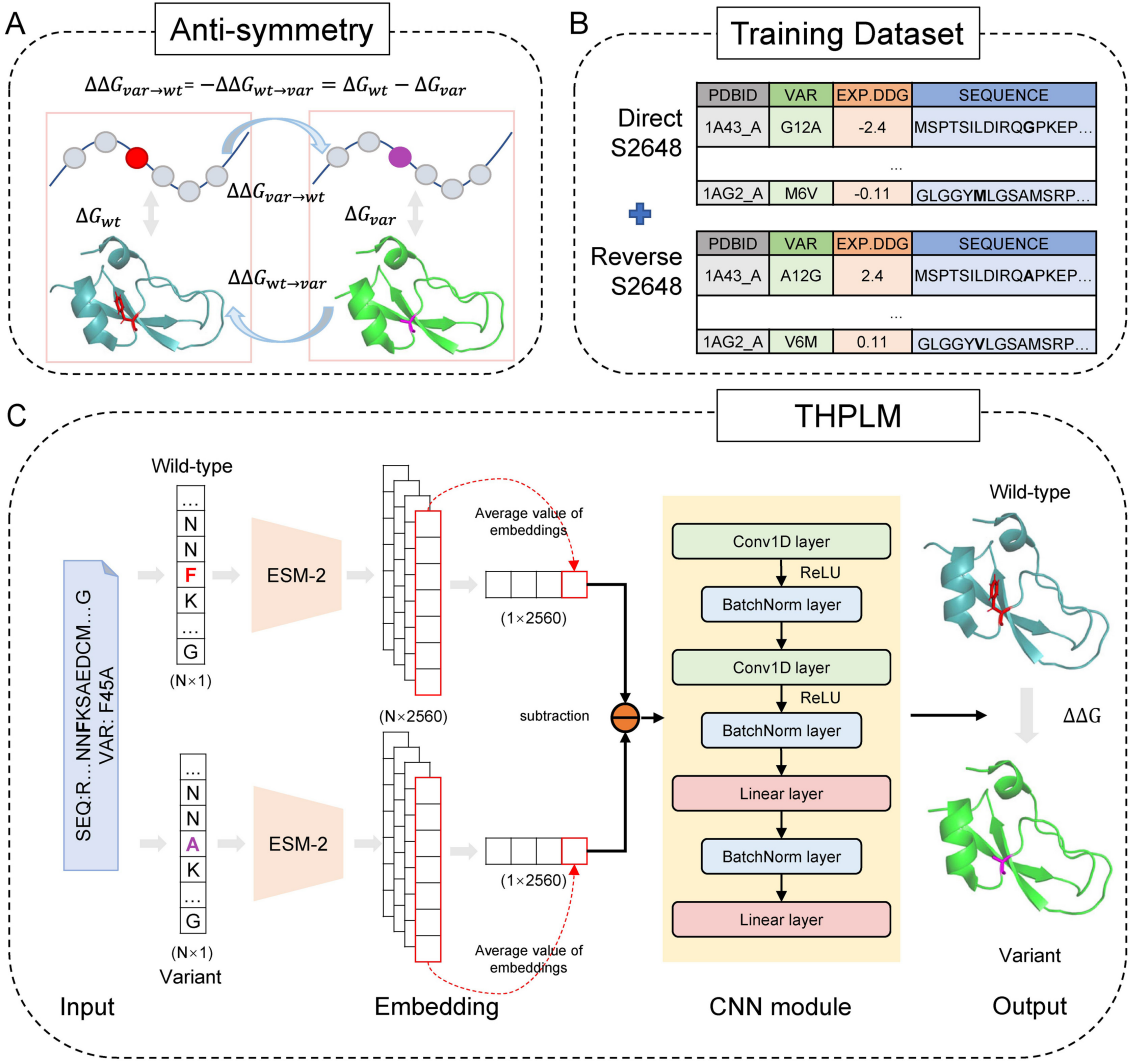
The framework of THPLM. (A) The definition of ΔΔ*G* and variation antisymmetry. (B) We used S2648 as training dataset for model training, including direct and reverse variations. (C) The overview of THPLM architecture. THPLM requires wild-type sequence and variant as input. ESM-2 outputs representations with *N* × 2560, where 2560 is the dimension of embeddings and *N* is the length of sequences. Subtraction between embeddings averaged over the entire sequence of wild-type and variant was conducted and passed into the CNN decoder module. The output of THPLM is the predicted value of ΔΔG.

Four datasets were collected from ProTherm (Kumar *et al.* 2006, Nikam *et al.* 2021) Databases including S2648, S669 (Pancotti *et al.* 2022), S^sym^ (Pucci *et al.* 2018), and CAGI5 Challenge’s Frataxin (Fowler and Fields 2014, Pancotti *et al.* 2021). As shown in Fig. 1A, the ΔΔ*G* of direct variation and reverse variation is antisymmetric. To build an antisymmetric model, we used the combination of direct and reverse S2648 variations as training datasets (Fig. 1B), with a random split of 9:1. To perform cross-validation, we used homology-based cross-validation split of S2648 by authors (Fariselli *et al.* 2015).

S669, S^sym^148, and Frataxin were used as testing datasets. S669 is from the newly updated ProThermDB (Nikam *et al.* 2021), and variants in S669 have less than 25% sequence identity with those in S2648. As for S^sym^ and Frataxin, variations present in S2648 were removed (Supplementary Figs S1 and S2). 296 variations in S^sym^ (S^sym^148) and 16 variations in Frataxin remained.

Moreover, S350, the subset of S2648, which is widely used for model evaluation, was also used to assess the performance of the neural network framework. We used the rest 2298 variations from S2468 to train a new model to make an objective performance comparison. The detailed information of variations in four datasets is described in Supplementary Tables S2 and S3.

### 2.2 Overview of THPLM

THPLM is an encoder–decoder architecture that converts wild-type and mutant protein sequences into representations and then uses convolutional and fully connected layers for the regression task (ΔΔ*G* prediction). It is an end-to-end model which requires amino acid sequences and single-point variations as inputs to predict the value of ΔΔ*G*. In Fig. 1C, the ESM-2 in encoder module was directly used as a Python library and did not participate in the backpropagation process. The decoder comprises two convolutional layers and two fully connected layers for training and predicting ΔΔ*G* (detailed training process is described in the Supplementary Materials).

#### 2.2.1 Protein language model ESM-2

The Meta Fundamental AI Research Team has released multiple versions of pretrained PLMs including ESM-2 (Lin *et al.* 2023), ESM-1b (Rives *et al.* 2021), ESM-MSA-1b (Rao *et al.* 2021), and ESM-1v (Meier *et al.* 2021) models. Newly released ESM-2 outperforms previous ESM models and others PLMs like Prot-T5-XL (Elnaggar *et al.* 2022) and CARP (Yang *et al.* 2022) on structure prediction. Therefore, we used ESM-2 to generate representations of wild-type protein and variant. As the parameters of the ESM-2 model increase, the embedding dimension increases, indicating that the protein representation will be more informative (Lin *et al.* 2023). At the same time, hardware requirements become higher. Considering the models’ performance and the hardware limitation, we used the pretrained model with 3 billion parameters (esm2_t36_3B_UR50D) as the embedding layer. In Fig. 1C, ESM-2 outputs the protein representation with the dimension of *N* × 2560, where 2560 is the dimension of embeddings and *N* is the length of amino acid sequence. Next, THPLM calculates the mean value for each embedding to reduce the impact of each amino acid position on the overall stability information. Then, subtraction between embeddings of wild-type and variant is conducted and passed into the CNN decoder module.

#### 2.2.2 Convolution neural network module

The convolution neural network (Smirnov *et al.* 2014) (CNN) module is constructed by two 1D convolution (Conv1D) layers and two linear layers with batch normalization (BatchNorm) and Rectified Linear Unit (ReLU) activate function, as shown in Fig. 1C (CNN module).

The output value of two Conv1D layers is described as:

$$E_{2}=\mathrm{BN}\left(\sigma \left(w_{2}*\mathrm{BN}\left(\sigma \left(w_{1}*{\mathrm{Feat}}_{wt\to \mathrm{var}}+b_{1}\right)\right)+b_{2}\right)\right),$$

where ${\mathrm{Feat}}_{wt\to \mathrm{var}}$ is a subtraction between embeddings of wild-type and variant. σ is the ReLU activate function. BN is the BatchNorm function. $w_{1}$, $w_{2},\;b_{1}$, and $b_{2}$ are the learnable parameters.

The output value of the CNN module is defined as:

$$\hat{Y}=\mathrm{Linear}\left(w_{4}*\mathrm{BN}\left(\mathrm{Linear}\left(w_{3}*\mathrm{Flatten}\left(E_{2}\right)+b_{3}\right)\right)+b_{4}\right),$$

where Flatten represents that $E_{2}$ is flattened to one dimension; $w_{3}$, $w_{4}$, $b_{3}$, and $b_{4}$ are the learnable parameters as well.

### 2.3 Evaluation metrics

We used five metrics to assess the performance of method THPLM, including PCC, root mean squared error (RMSE), and accuracy (ACC) between the predicted ΔΔGs and experimental ΔΔGs, as well as antisymmetric property (PCC) and bias property (<δ>) between the predicted ΔΔGs of the direct and corresponding reverse variants. The formulations are shown in the Supplementary Materials.

## 3 Results

In this study, we proposed THPLM, a deep learning model for ΔΔG prediction. Unlike existing sequence-based methods that directly perform supervised learning regression on sequences, THPLM first generated representations of protein sequences using PLM, enabling the model to exploit protein structural information and understand how point variations lead to stability changes (Meier *et al.* 2021). In THPLM architecture, we used the pretrained PLM ESM-2 from Meta AI Research to automatically generate representations for wild-type and variant sequences. To verify whether the PLM can effectively extract information about stability changes, we made UMAP plots of subtraction between variant and wild-type sequences classified by negative or positive ΔΔGs (Supplementary Fig. S3). The result suggested that using PLM (Section 2, Fig. 1C) is feasible and reasonable.

### 3.1 Performance on multiple testing datasets

#### 3.1.1 Cross-validation on S2648

Since the training data are small, overfitting is one of the major concerns in our study. We then used homology-based cross-validation split of S2648, which is grouped by sequence identity to remove the bias from similarity between training and testing sets, to perform 5-fold cross-validation (Fariselli *et al.* 2015). We ensembled five cross-validation models by averaging method and named it THPLM^E^. The performance of five cross-validation models is shown in Supplementary Table S4. The mean PCCs for the direct, reverse, and overall variations were 0.76, 0.77, and 0.86, and the mean ACCs for the direct, reverse, and overall variations were 0.86, 0.86, and 0.87, respectively.

#### 3.1.2 Performance on three testing datasets

To demonstrate the robustness and generalization of THPLM, we evaluated the model performance on three testing datasets including S^sym^148, S669, and Frataxin. We used the PCC and RMSE to assess the model’s performance in estimating ΔΔ*G*. Additionally, we used accuracy (ACC) to evaluate the model in distinguishing stabilizing and destabilizing variations. The results are shown in Table 1. In S^sym^148, THPLM achieved PCCs of 0.76 for direct variations, 0.76 for reverse variations, and 0.83 for overall variations. The ACCs of classification are 0.84, 0.82, and 0.83 in direct, reverse, and overall variations, respectively. In the S669 testing dataset, THPLM only achieved PCCs of 0.39 for direct variations, 0.35 for reverse variations, and 0.53 for overall variations, much lower than PCCs in S^sym^148. However, the ACC values are acceptable, which are 0.74, 0.73, and 0.74, respectively. In the Frataxin testing dataset, the PCCs between predicted ΔΔ*G* and experimental ΔΔ*G* are 0.80 for overall variations, 0.67 for direct variations, and 0.72 for reverse variations. However, RMSE reached around 3.55 Kcal/mol, which indicated a specific difference between the predicted and the experimental values.

**Table 1.** Performance of THPLM on S^sym^148, S669, and Frataxin testing datasets.

	Direct			Reverse			Direct + reverse			Bias/Antisymmetry	
	PCC	ACC	RMSE	PCC	ACC	RMSE	PCC	ACC	RMSE	PCC	<δ>
S^sym^148	0.76	0.84	1.24	0.76	0.82	1.25	0.86	0.83	1.25	−1.00	−0.02
S669	0.39	0.74	1.60	0.35	0.73	1.66	0.53	0.74	1.63	−0.96	−0.01
Frataxin	0.67	0.62	3.55	0.72	0.75	3.55	0.80	0.69	3.55	−1.00	−0.02

**Figure 2.**
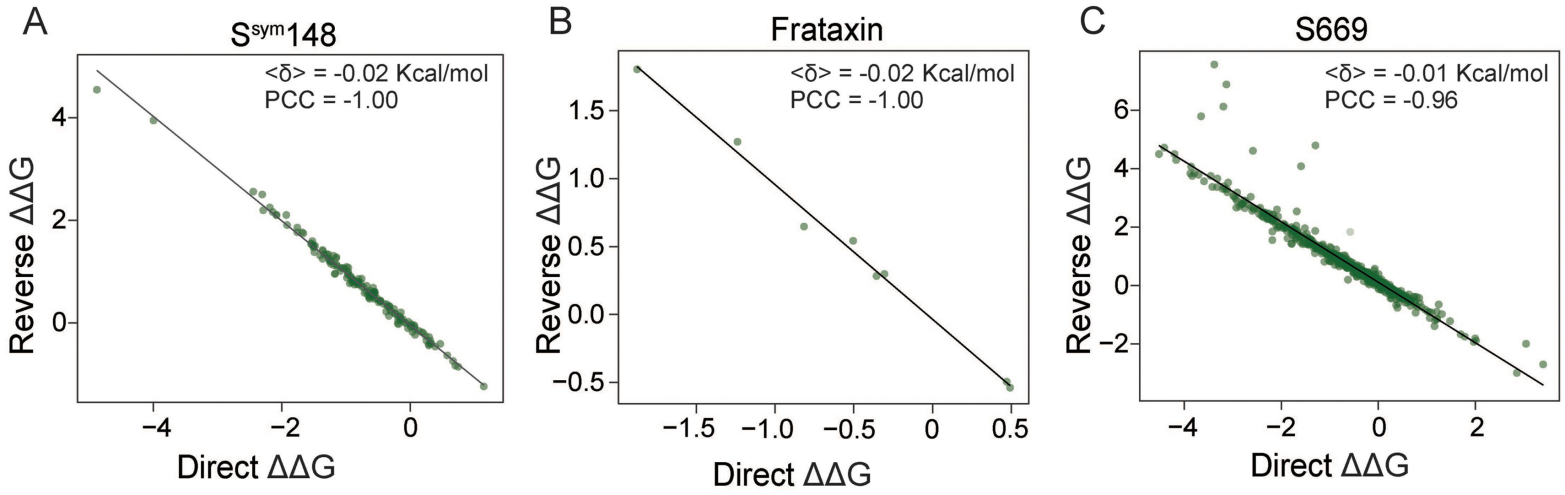
The antisymmetric properties of THPLM on S^sym^148, S669, and Frataxin testing datasets. *X*-axis represents predicted ΔΔ*G*s of direct variations, and *Y*-axis represents predicted ΔΔ*G*s of reverse variations. (A) S^sym^148 alone (PCC = −1.00, <*δ*> = −0.02 Kcal/mol); (B) Frataxin alone (PCC = −1.00, <*δ*> = −0.02 Kcal/mol); (C) S669 alone (PCC = −0.96, <*δ*> = −0.01 Kcal/mol).

As shown in Fig. 2A–C, the antisymmetric property on datasets S^sym^148, Frataxin, and S669 are closest to −1. The PCC between direct S669 variations and their reverse variations is −0.96. The 〈*δ*〉 for the S669 dataset is close to 0 (−0.01 Kcal/mol). The results demonstrate that our method THPLM is a predictor with higher antisymmetric properties and without bias.

### 3.2 Performance comparison with other sequence-based methods

#### 3.2.1 Performance comparison on three S^sym^148, S669, and Frataxin testing datasets

**Figure 3.**
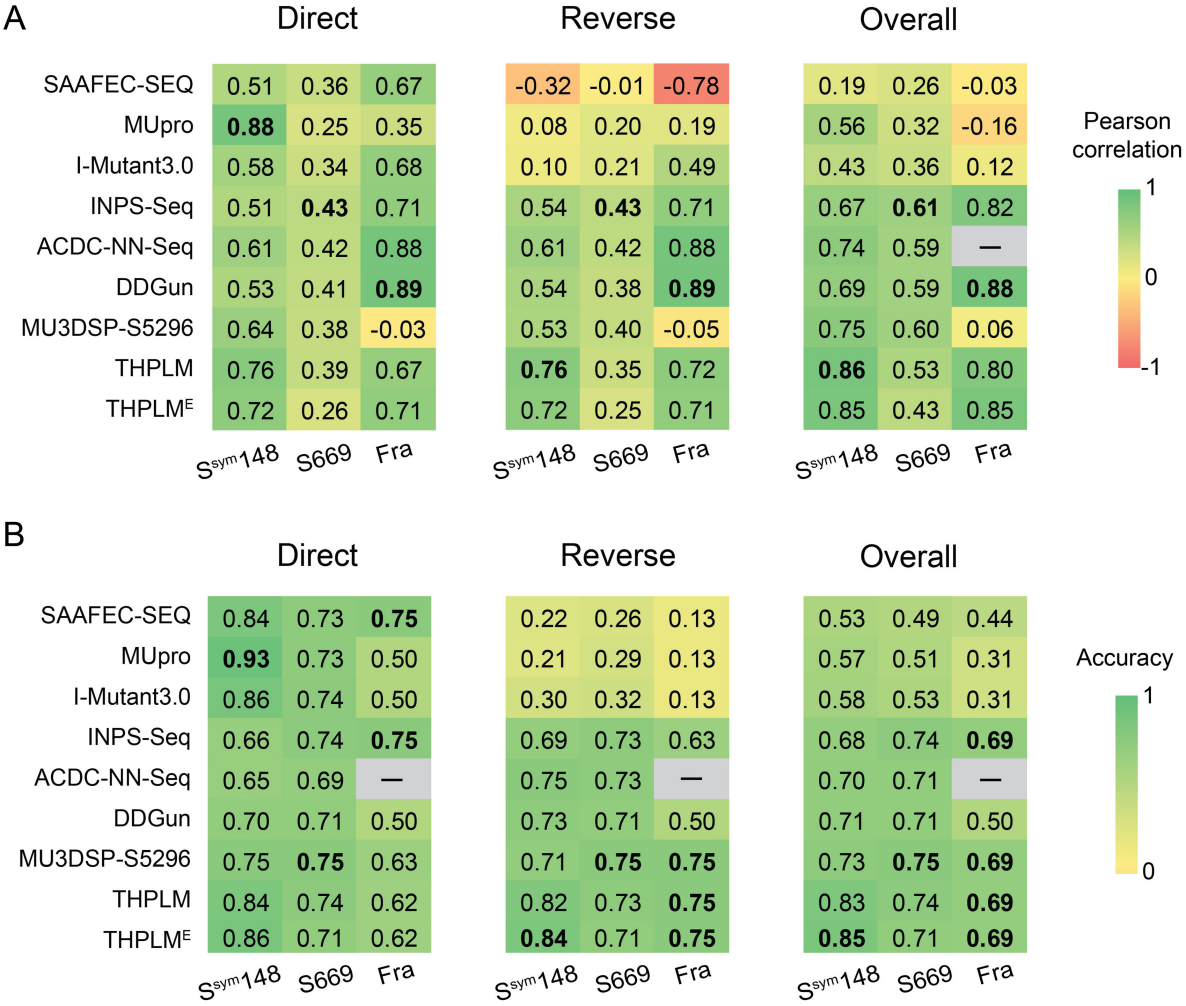
Performance comparison among sequence-based methods on S^sym^148, S669, and Frataxin testing datasets. (A) THPLM achieved PCCs of 0.86, 0.53, and 0.80 for overall variations in three testing datasets. (B) THPLM achieved ACCs of 0.83, 0.74, and 0.69 for overall variations in three testing datasets. “*” represents results from the subset of S669 due to the missing predictions. “–” means no result from ACDC-NN-Seq due to an incorrect profile. Fra: Frataxin.

For comparison with other sequence-based methods, seven state-of-the-art methods were performed on three testing datasets, including INPS(INPS-Seq) (Fariselli *et al.* 2015), ACDC-NN-Seq (Pancotti *et al.* 2021), DDGun (Montanucci *et al.* 2019), I-Mutant3.0 (Capriotti *et al.* 2005), MUpro (Cheng *et al.* 2006), MU3DSP (Gong *et al.* 2023), and SAAFEC-SEQ (Li *et al.* 2021). Among them, INPS, ACDC-NN-Seq, and DDGun consider the ΔΔ*G* prediction of direct and reverse variations, while I-Mutant3.0, MUpro, and SAAFEC-SEQ only consider the ΔΔ*G* prediction of direct variations. Comparative performance of THPLM and THPLM^E^ on S^sym^148, S669, and Frataxin with other sequence-base predictors was shown in Fig. 3.

In testing dataset S^sym^148, THPLM achieved the best prediction performance on the reverse and overall variations, significantly higher than the performance of other methods. Considering direct variations alone, only MUpro performed better than THPLM, with a PCC of 0.88 (ACC = 0.93 and RMSE = 0.74; Supplementary Table S5), indicating that THPLM is better for the antisymmetric task. The PCC values obtained by all sequence-based methods showed a significant decline. There may exist a data shift in the distribution of ΔΔ*G* values between S669 and S2648. Thus, we used ACC rather than PCC and RMSE to assess the performance of all methods. In testing dataset S669, THPLM has a comparable performance with other methods in terms of ACC. Among them, MU3DSP-S5296 has the best ACC of 0.75 (THPLM: 0.74), 0.75 (THPLM: 0.73), and 0.75 (THPLM: 0.74) in the prediction of direct variations, reverse variations, and overall variations, respectively. However, the antisymmetric and bias properties of MU3DSP-S5296 do not exceed our model THPLM. As for testing dataset Frataxin, the RMSE values of the prediction results of all methods have increased to a certain extent, ranging from 2.25 (DDGun for reverse variations) to 5.11 Kcal/mol (SAAFEC-SEQ for reverse variations). In terms of PCCs, DDGun outperformed other methods, reaching 0.89 for both direct and reverse variations, and 0.88 for overall variations. However, the ACCs of DDGun were only 0.50. THPLM and THPLM^E^ achieved the best ACCs of 0.75 (reverse) and 0.69 (overall), respectively.

#### 3.2.2 Performance comparison on S350 testing dataset

**Figure 4.**
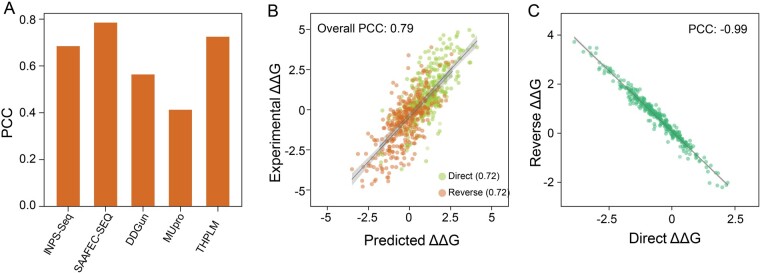
The performance of retrained THPLM on S350 testing dataset. (A) Performance comparison on direct variations of S350. THPLM achieved a PCC of 0.72. (B) Performance of THPLM on both direct and reverse variations of S350, with an overall PCC of 0.79. (C) The antisymmetric properties of THPLM on S350. PCC equals to −0.99 and <δ> equals to 0.03 Kcal/mol.

Since S350 is a subset of the S2648 training set and widely used for performance comparison, we retrained the model by removing S350 from the S2648 to make a comprehensive comparison. Considering that many compared predictors can’t predict the reverse variations, such as SAEEFC-SEQ and MUpro, THPLM was compared with sequence-based predictors including DDGun, INPS, SAEEFC-SEQ, and MUpro on the direct S350 dataset. THPLM was the second-best method (SAEEFC-SEQ, PCC = 0.78), and PCC on S350 in total achieved 0.79 (Fig. 4A). The retrained THPLM reached PCCs of 0.72 for both direct and reverse variations of S350 dataset and 0.79 for overall (Fig. 4B). The antisymmetric property on predicting S350 is nearly perfect which PCC equal to −0.99 (Fig. 4C). The performance demonstrates that THPLM has the ability to predict stability changes.

### 3.3 Performance comparison with other structure-based methods

We compared structure-based methods on the S^sym^148 testing dataset, as THPLM and THPLM^E^ used pretrained PLM which might contain atomic-level structure information and inner structure interaction information. Model1426 was trained by the rest variations after removing over 25% sequence identity with S^sym^ from dataset S2648. The structure-based methods include FoldX (Guerois *et al.* 2002), PremPS (Chen *et al.* 2020), Dynamut2 (Rodrigues *et al.* 2021), mCSM (Pires *et al.* 2014), SDM (Worth *et al.* 2011), DUET (Pires *et al.* 2014), ACDC-NN (Benevenuta *et al.* 2021), PoPMuSiC (Pucci *et al.* 2018), DDGun3D (Montanucci *et al.* 2019), INPS3D (Savojardo *et al.* 2016), Dynamut (Rodrigues *et al.* 2018), and Rosetta (Kellogg *et al.* 2011).

Table 2 shows each method’s obtained performance, bias, and antisymmetric properties. THPLM^E^ outperformed all structure-based methods in terms of ACCs, and RMSEs except for the PCCs of THPLM. THPLM achieved the best performance of PCCs with 0.76, 0.76, and 0.86 on direct, reverse, and overall variations. Model1426, which was trained using the THPLM framework and used proteins with low sequence similarity, achieved a PCC of 0.82 and an ACC of 0.80 on overall variations. In addition, both THPLM and THPLM^E^ exhibited good antisymmetry, with a −1.00/−0.02 (PCC/<δ>) and −1.00/0.00 Kcal/mol (PCC/<δ>), respectively.

**Table 2.** Performance comparison of THPLM with twelve structure-based methods on dataset S^sym^148.

Method	Direct			Reverse			Direct + reverse			Bias/Antisymmetry	
	PCC	ACC	RMSE	PCC	ACC	RMSE	PCC	ACC	RMSE	PCC	<δ>
FoldX	0.47	0.76	2.40	0.30	0.57	2.56	0.45	0.66	2.48	−0.08	−0.77
PremPS	0.72	0.81	1.20	0.64	0.82	1.34	0.83	0.82	1.27	−0.92	−0.07
Dynamut	0.50	0.66	1.65	0.33	0.66	1.97	0.48	0.66	1.82	−0.52	−0.15
mCSM	0.55	0.78	1.33	0.03	0.24	2.78	0.35	0.51	2.18	−0.26	−1.00
SDM	0.49	0.78	1.69	0.10	0.43	2.69	0.30	0.60	2.25	−0.49	−0.63
DUET	0.59	0.82	1.32	0.07	0.36	2.63	0.40	0.59	2.08	−0.30	−0.84
ACDC-NN-str	0.64	0.73	1.53	0.64	0.74	1.53	0.76	0.73	1.53	−1.00	0.00
PoPMuSiC	0.62	0.84	1.27	0.22	0.34	2.49	0.51	0.59	1.97	−0.35	−0.82
DDGun3D	0.58	0.71	1.49	0.54	0.72	1.54	0.68	0.72	1.51	−0.99	−0.02
INPS3D	0.61	0.82	1.30	0.28	0.41	2.24	0.57	0.62	1.83	−0.52	−0.62
Dynamut2	0.57	0.76	1.33	−0.04	0.26	2.77	0.33	0.51	2.17	−0.10	−0.89
Rosetta	−0.12	0.46	28.33	−0.12	0.48	28.33	−0.18	0.47	28.33	−1.00	0.00
Model1426	0.66	0.81	1.35	0.67	0.78	1.35	0.82	0.80	1.35	−0.99	−0.01
THPLM	**0.76**	0.84	1.24	**0.76**	0.82	1.25	**0.86**	0.83	1.25	−1.00	−0.02
THPLM^E^	0.72	**0.86**	**1.10**	0.72	**0.84**	**1.09**	0.85	**0.84**	**1.09**	−1.00	0.00

The best results in each category are marked in bold.

### 3.4 Case study: the effect of A172L variation on the stability of 3-isopropylmalate dehydrogenase

**Figure 5.**
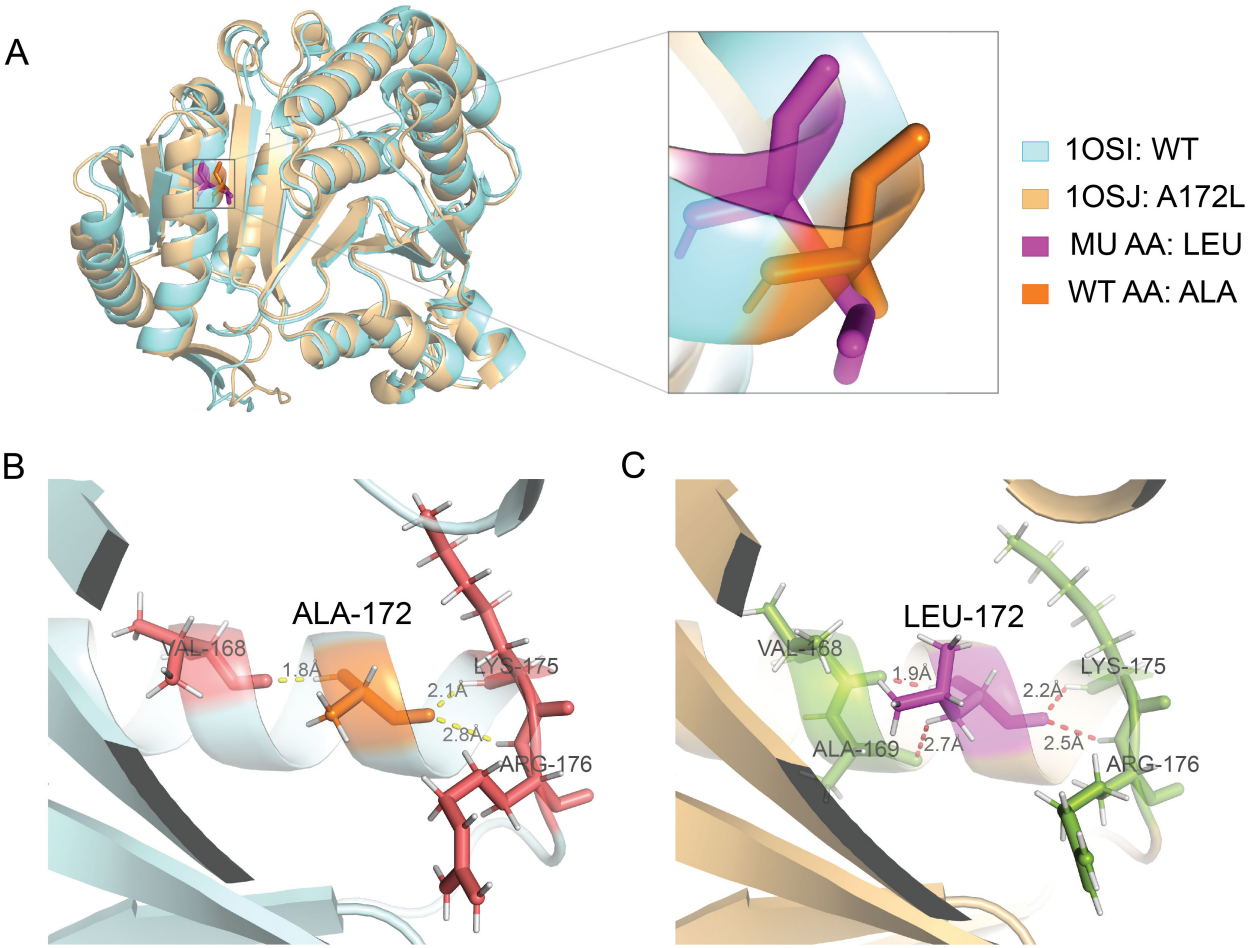
Interpretation of A172L variations of 3-isopropylmalate dehydrogenase. (A) Aligned structures of WT 3-isopropylmalate dehydrogenase (light blue) and A172L variant (wheat). MU AA: mutated amino acid, WT AA: wild-type amino acid. (B) Cut-away view of residue ALA-172 on WT 3-isopropylmalate dehydrogenase with Hydrogen bonds (H-bond) interactions with residues LYS-175, ARG-176, and VAL-168. (C) Cut-away view of residue LEU-172 on mutated 3-isopropylmalate dehydrogenase with Hydrogen bonds (H-bond) interactions with residues LYS-175, ARG-176, ALA-169, and VAL-168.

As a case study, we chose the 3-isopropylmalate dehydrogenase with experimental recorded structure before and after variation. 3-Isopropylmalate dehydrogenase (E.C. 1.1.1.85) is coded by the *leuB* gene from *Thermus thermophilus* strain HB8, and expressed in *Escherichia coli* carrying a recombinant plasmid (Hobbs *et al.* 2012). We collected the wild-type structure (PDB: 1OSI) and ALA172LEU (A172L) variant structure (PDB: 1OSJ) (Yamada *et al.* 1990) and used THPLM to predict the effect of A172L on the stability changes. In Fig. 5A, the A172L is located at the interface between domains and the $C_{\alpha }$ trace of the mutant structure differs from the wild-type structure by 1.7 Å (Hobbs *et al.* 2012). With Residue Interaction Network Generator (version 3.0) (Clementel *et al.* 2022), we found that the A172L variant has one more hydrogen bond (ALA-169, Fig. 5B and C) and two more Van Der Waals interactions (VAL-168 and VAL-131, Supplementary Table S6). The result shows that the A172L is a stabilizing variation. Besides that, the study (Yamada *et al.* 1990) shows that the substitution of LEU enhances the local filling of side chains to transfer the main chains of opposite domains and contributes to the thermal stability of the mutated protein. THPLM and THPLM^E^ predicted that the A172L is a stabilizing variation with the ΔΔ*G* of 0.77 and 0.69 Kcal/mol, respectively, consistent with the experimental conclusions.

## 4 Discussion

With advances in protein structure prediction, pretrained PLMs have shown promising potential in addressing protein-related issues. Pretrained PLMs can reflect atomic-level structural information from amino acid sequences, effectively improving the performance of sequence-based tools. Here, we developed a new sequence-based method, THPLM, to predict stability changes of the point variation using ESM-2. With two convolutional layers and two fully connected layers, THPLM and THPLM^E^ achieved comparable or even better performance than 7 sequence-based and 12 structure-based methods and can satisfy the antisymmetric property. Meanwhile, THPLM and THPLM^E^ correctly predicted the effect of variation A172L on the stability of 3-isopropylmalate dehydrogenase.

This study used three testing datasets from different studies to make a comprehensive assessment. Duplicated variations between training and testing datasets were removed for objective model evaluation. Our model outperformed all other methods on the S^sym^148 dataset and achieved comparable performance to sequence-based methods on the S669 and Frataxin sets. Interestingly, we found that the performance of all sequence-based methods on the S669 and Frataxin datasets is worse than that on S^sym^148, with lower PCC and higher RMSE values. This may be due to the diversity of training data or the deviation in the measurement of experimental ΔΔ*G*, so we used ACC to compare the performance of all methods on classification (stabilizing or destabilizing). Furthermore, the predicting results of the S^sym^148 dataset achieved a better performance than that of S^sym^ (Supplementary Table S7). This might be due to the fact that the predicting results for some proteins in the S^sym^ dataset are not satisfactory (such as 1EY0 and 2RN2 in Supplementary Table S8). All in all, the framework THPLM demonstrated comparable robustness, as it maintained high accuracy under various conditions and scenarios, especially on low-homology sequences.

However, there are some limitations to our study. First, the THPLM performance is limited by the training dataset’s scaling and diversity (Nikam *et al.* 2021, Stourac *et al.* 2021). Second, we unconsidered multipoint variations in this study, though we believe that using PLM may provide a promising way to predict the effect of multipoint variations.

In general, THPLM successfully predicted stability changes of single-point variations only using sequences. The performance of THPLM demonstrated that the embeddings from the PLM contributed to discovering the minor changes in protein after variations. Hopefully, more data on protein stability changes will be available for further research in the future.

## Supplementary Material

btad646_Supplementary_Data
