# Supplementary material for: THPLM: a sequence-based deep learning framework for protein stability changes prediction upon point variations using pretrained protein language model
Source: Bioinformatics. 2023 Oct 24;39(11):btad646. doi: 10.1093/bioinformatics/btad646 (PMC10627365; doi:10.1093/bioinformatics/btad646)
Supplement: btad646_Supplementary_Data [file btad646_supplementary_data.docx]

# Supplementary materials

## Methods used for performance comparison

We assessed the performance of THPLM and compared it with seven sequence-based and twelve structure-based methods. Sequence-based methods include INPS (Fariselli, et al., 2015), ACDC-NN-seq (Pancotti, et al., 2021), DDGun (Montanucci, et al., 2019), I-Mutant3.0 (Capriotti, et al., 2005), MUpro (Cheng, et al., 2006), MU3DSP (Gong, et al., 2023), and SAAFEC-SEQ (Li, et al., 2021). Among them, ACDC-NN-Seq can’t predict variants from Frataxin dataset because the profile file can’t be generated correctly. The PCCs of ACDC-NN-Seq on direct and reverse Frataxin datasets are from the research literature (Pancotti, et al., 2021). Besides, there is no result for some reverse variations in dataset S669 using SAAFEC-SEQ and DDGun. Thus, we obtained the PCCs from the research literature (Pancotti, et al., 2022), while the ACCs were calculated manually. The number of instances used for calculating ACCs of SAAFEC-SEQ is 660 of 669 and of DDGun is 642 of 669. Structure-based methods include FoldX (Guerois, et al., 2002), PremPS (Chen, et al., 2020), Dynamut2 (Rodrigues, et al., 2021), mCSM (Pires, et al., 2014), SDM (Worth, et al., 2011), DUET (Pires, et al., 2014), ACDC-NN (Benevenuta, et al., 2021), PoPMuSiC (Pucci, et al., 2018), DDGun3D (Montanucci, et al., 2019), INPS3D (Savojardo, et al., 2016), Dynamut (Rodrigues, et al., 2018) and Rosetta (Kellogg, et al., 2011). We made a summary of current methods that were shown in Supplementary Table 1.

## Evaluation metrics

We used five metrics to assess and compare the performance of different methods, including Pearson Correlation Coefficient (PCC), Root Mean Squared Error (RMSE), accuracy (ACC) between experimental $\Delta\Delta G$s and predicted $\Delta\Delta G$s, and bias and antisymmetric properties (PCC, $\left\langle\delta\right\rangle$) of the free energy changes between wild-type and variants. The definitions are shown below:

$$PCC=\frac{cov\left( \hat{Y},Y \right)}{\sigma_{\hat{Y}}\sigma_{Y}}$$

$$RMSE= \sqrt{\frac{1}{N}\sum_{i=1}^{N} \left( Y_{i}-\hat{Y}_{i} \right)^{2}}$$

where $\hat{Y}$ and $Y$ represent $\Delta\Delta G$ from prediction and experiment, respectively; $cov\left( \hat{Y},Y \right)$ is the covariance of $\hat{Y}$ and $Y$; $\sigma_{\hat{Y}}$ and $\sigma_{Y}$ represent the standard deviation of the variables $\hat{Y}$ and $Y$, respectively. $N$ represents the total number of instances.

To make a basic comparison among methods, we implemented ACC to assess the performance of the model on the identification of stabilizing and destabilizing variations in proteins:

$$ACC=\frac{TP+TN}{N}$$

where $TP$, $TN$, and $N$ are the number of true positive, true negative and instances, respectively. $TP$ means that both experimental result and model prediction suggest this is a stabilizing variation, while TN for the destabilizing variation.

Since some methods were trained on a set of unbalanced data where most variations were destabilizing, the bias property of each method was obtained by measuring the average bias between direct and reverse $\Delta\Delta G$ of the same variation. The average bias is denoted as $\left\langle\delta\right\rangle$:

$$\left\langle\delta\right\rangle=\frac{\sum_{i=0}^{N} \left( \Delta\Delta G_{i}^{dir}+\Delta\Delta G_{i}^{rev} \right)}{2N}$$

Furthermore, we also used antisymmetric properties (PCC) between the predicted $\Delta\Delta G$s of the direct and corresponding reverse variations. In general, a perfect antisymmetric method should make $\left\langle\delta\right\rangle$equal 0 and PCC equal to -1.

## Training process

The model is built and trained with PyTorch 1.12.1 and Python 3.8.13. We used Adam (Kingma and Ba, 2014) as the optimizer and MSE as the loss function. The learning rate is set to 5×10^-4^. Early stopping was used during the training process to avoid the problem of model overfitting. In detail, if the model loss on the validation set had continuously increased for 100 epochs, we stopped the training process and went back to find the optimal model weights. The detailed hyperparameters of THPLM are shown in Supplementary Table 9.

The loss function is mean squared error (MSE):

$${Loss}_{MSE}\left( y_{i}\left( x \right),\hat{y}_{i}\left( x \right) \right)= \frac{1}{N}\sum_{i=1}^{N} \left( y_{i}\left( x \right)-\hat{y}_{i}\left( x \right) \right)^{2},$$

where $N$ is the number of instances, $y_{i}\left( x \right)$ represents experimental $\Delta\Delta G$ and $\hat{y}_{i}\left( x \right)$represents predicted $\Delta\Delta G$.

**Cross-validation on S2648**

Since the training data is small, overfitting is one of the major concerns in our study. We used two methods to do cross-validation. One is replicated the 10-fold cross-validation 10 times with randomly re-generated folds and averaged the results on the training data S2648. The result showed the mean value of PCC reached 0.8, ranging from 0.77 to 0.82. RMSE ranges from 1.17 Kcal/mol to 0.98 Kcal/mol, and ACC ranges from 0.79 to 0.85 (Supplementary Figure 4). Another is used five-fold cross-validation by considering the sequence identity provided by authors (Fariselli, et al., 2015), the results are shown in supplementary Table 4. The average PCC is 0.76, 0.77, and 0.86 on direct, reverse, and overall variations, respectively. The average ACC is 0.86, 0.86, and 0.87 on direct, reverse, and overall variations, respectively.

# Supplementary Tables

**Supplementary Table 1**. List of methods starting from structure or sequence to predict the thermodynamic stability changes of proteins upon single-point mutations

| Methods (Year) | Algorithms | | Input (Structure or sequence) | | URLs | reference |
| --- | --- | --- | --- | --- | --- | --- |
| FoldX  (2002) | linear regression | | Structure | | <http://foldxsuite.crg.eu/> | (Guerois, et al., 2002) |
| SDM (2011) | Linear combi- nation | | Structure | | [Access by http://biosig.unimelb.edu.au/duet/stability](http://biosig.unimelb.edu.au/dynamut/) | (Worth, et al., 2011) |
| Rosetta  (2011) | Rosetta Energy | | Structure | | https://www.rosettacommons.org/software | (Kellogg, et al., 2011) |
| DUET (2014) | Integrated model (SVM) | | Structure | | <http://biosig.unimelb.edu.au/duet/stability> | (Pires, et al., 2014) |
| mCSM (2014) | Gaussian and random forest | | Structure | | <http://biosig.unimelb.edu.au/mcsm/> | (Pires, et al., 2014) |
| INPS3D (2016) | SVM | | Structure | | [http://inps.biocomp.unibo.it](http://inps.biocomp.unibo.it/) | (Savojardo, et al., 2016) |
| MAESTRO  (2016) | | ANN agents and SVM | | Structure | <https://biwww.che.sbg.ac.at/maestro/web> | (Laimer, et al., 2015) |
| SDM2 (2017) | Linear combination | | Structure | | <http://marid.bioc.cam.ac.uk/sdm2/prediction> | (Pandurangan, et al., 2017) |
| PoPMuSiC^sym^  (Pucci, et al., 2018) (2018) | Neural network | | Structure | | <https://soft.dezyme.com/query/create/pop> | (Pucci, et al., 2018) |
| DynaMut  (2018) | Integrated model | | Structure | | <http://biosig.unimelb.edu.au/dynamut/>. | (Rodrigues, et al., 2018) |
| DDGun3D  (2019) | Linear parametric model | | Structure | | <https://github.com/biofold/ddgun> | (Montanucci, et al., 2019) |
| DynaMut2 (2020) | Random forest | | Structure | | <http://biosig.unimelb.edu.au/dynamut2/> | (Rodrigues, et al., 2021) |
| ACDC-NN  (2021) | CNN | | Structure | | https://github.com/compbiomed-unito/acdc-nn | (Benevenuta, et al., 2021) |
| PremPS (2020) | Random forest | | Structure | | <https://lilab.jysw.suda.edu.cn/research/PremPS/> | (Chen, et al., 2020) |
| MUpro (2006) | SVM | | Sequence | | https://www.ics.uci.edu/~baldig/mutation.html | (Cheng, et al., 2006) |
| EASE-MM (2016) | Integrated models based SVM | | Sequence | | https://www.sparks-lab.org/server/ease-mm/ | (Folkman, et al., 2016) |
| INPS  (2016) | SVM | | Sequence | | http://inps.biocomp.unibo.it | (Fariselli, et al., 2015) |
| DDGun (2019) | Linear  parametric  model | | Sequence | | https://github.com/biofold/ddgun | (Montanucci, et al., 2019) |
| SAAFEC-SEQ (2021) | Gradient boosting | | Sequence | | <http://compbio.clemson.edu/SAAFEC-SEQ/index.php> | (Li, et al., 2021) |
| ACDC-NN-Seq  (2021) | CNN | | Sequence | | https://github.com/compbiomed-unito/acdc-nn | (Pancotti, et al., 2021) |
| MU3DSP  (2023) | LightGBM | | Sequence | | https://github.com/hurraygong/MU3DSP | (Gong, et al., 2023) |
| I-Mutant 2.0 (2005) | SVM | | Structure or sequence | | https://folding.biofold.org/cgi-bin/i-mutant2.0.cgi | (Capriotti, et al., 2005) |

SVM: Support Vector Machines; ANN: Artificial Neural Network; CNN: Convolutional Neural Networks; LightGBM: Light Gradient Boosting Machine.

**Supplementary Table 2.** Datasets used to build, evaluate, and independently test in THPLM and THPLM^E^

| Dataset  (direct + reverse) | Total Variations | Direct Variations | Reverse Variations | Additional Details |
| --- | --- | --- | --- | --- |
| S2648 | 5296 | 2648 | 2648 | Unique Variations/Averaged ΔΔG |
| S2298 | 4596 | 2298 | 2298 | Unique Variations/Averaged ΔΔG |
| S1426 | 2852 | 1426 | 1426 | Unique Variations/Averaged ΔΔG |
| S350 | 700 | 350 | 350 | Unique Variations/Averaged ΔΔG |
| S^sym^ | 684 | 342 | 342 | Unique Variations |
| S^sym^148 | 296 | 148 | 148 | Unique Variations |
| S669 | 1338 | 669 | 669 | ∼20% of the ΔΔG changed |
| Frataxin | 16 | 8 | 8 | One Protein |

All datasets used in main document are direct + reverse variations.

**Supplementary Table 3.** The composition of total, **destabilizing**, **stabilizing, and neutral variations** of the original datasets in **Table 2**

| **Dataset** | **Total Variations (Proteins)** | **Destabilizing Variations (Proteins)** | **Stabilizing Variations (Proteins)** | **Neutral Variations (Proteins)** | **Additional Details** |
| --- | --- | --- | --- | --- | --- |
| S2648 | 2648(131) | 1598(106) | 295(74) | 755(97) | Unique Variations/Averaged ΔΔG |
| S2298 | 2298(125) | 1407(103) | 246(66) | 650(91) | Unique Variations/Averaged ΔΔG |
| S350 | 350(67) | 192(51) | 54(24) | 105(43) | Unique Variations/Averaged ΔΔG |
| S1426 | 1426(113) | 862(89) | 185(60) | 379(80) | Unique Variations/Averaged ΔΔG |
| S^sym^ | 342(15) | 188(13) | 37(9) | 117(10) | Unique Variations |
| S^sym^(reverse) | 342(342) | 37(37) | 188(188) | 117(117) | Unique Variations |
| S^sym^148 | 148(7) | 89(5) | 10(4) | 49(3) | Unique Variations |
| S669 | 669(94) | 85(40) | 389(69) | 195(47) | ∼20% of the ΔΔG changed |
| Frataxin | 8(1) | 5(1) | 0 | 3 (1) | One Protein |

Stabilizing Variations ($\Delta\Delta G>0$.5), Destabilizing Variations ($\Delta\Delta G<-0.5$), and Neutral Variations ($-0.5<\Delta\Delta G<0.5$), respectively. "2648(131)" stands for total 2648 variations on 131 proteins.

**Supplementary Table 4.** The performances of five-fold cross-validation models separably and their averaging performance

|  | Direct | | | Direct | | | Direct + Reverse | | | Bias/Antisymmetry | |
| --- | --- | --- | --- | --- | --- | --- | --- | --- | --- | --- | --- |
|  | PCC | ACC | RMSE | PCC | ACC | RMSE | PCC | ACC | RMSE | PCC | <$\delta$> |
| Fold0 | 0.63 | 0.80 | 1.17 | 0.64 | 0.82 | 1.12 | 0.78 | 0.81 | 1.14 | -0.99 | 0.04 |
| Fold1 | 0.79 | 0.80 | 0.97 | 0.79 | 0.80 | 0.97 | 0.85 | 0.80 | 0.97 | -1.00 | 0.00 |
| Fold2 | 0.92 | 0.94 | 0.51 | 0.91 | 0.93 | 0.53 | 0.95 | 0.94 | 0.52 | -0.99 | 0.01 |
| Fold3 | 0.71 | 0.91 | 0.89 | 0.71 | 0.91 | 0.87 | 0.86 | 0.91 | 0.88 | -0.98 | 0.03 |
| Fold4 | 0.77 | 0.87 | 0.88 | 0.78 | 0.88 | 0.87 | 0.85 | 0.87 | 0.88 | -0.99 | 0.00 |
| Mean | 0.76 | 0.86 | 0.88 | 0.77 | 0.87 | 0.87 | 0.86 | 0.87 | 0.88 | -0.99 | 0.02 |

**Supplementary Table 5**. Comparative performance of THPLM with seven sequence-based methods on dataset S^sym^148

| Method | Direct | | | Reverse | | | Direct + reverse | | | | Bias/Antisymmetry | | |
| --- | --- | --- | --- | --- | --- | --- | --- | --- | --- | --- | --- | --- | --- |
|  | PCC | ACC | RMSE | PCC | ACC | RMSE | PCC | ACC | RMSE | PCC | | <$\delta$> |  |
| I-Mutant3.0-Seq | 0.58 | 0.86 | 1.37 | 0.10 | 0.30 | 2.46 | 0.43 | 0.58 | 1.99 | -0.45 | | -0.68 |  |
| MUpro | 0.88 | 0.93 | 0.74 | 0.08 | 0.21 | 2.83 | 0.56 | 0.57 | 2.07 | -0.13 | | -1.15 |  |
| ACDC-NN-Seq | 0.61 | 0.65 | 1.52 | 0.61 | 0.75 | 1.53 | 0.74 | 0.70 | 1.52 | -1.00 | | 0.00 |  |
| INPS-Seq | 0.51 | 0.66 | 1.61 | 0.54 | 0.69 | 1.57 | 0.67 | 0.68 | 1.59 | -0.99 | | 0.01 |  |
| DDGun | 0.53 | 0.70 | 1.50 | 0.54 | 0.73 | 1.51 | 0.69 | 0.71 | 1.51 | -1.00 | | -0.01 |  |
| MU3DSP-S5296 | 0.64 | 0.75 | 1.52 | 0.53 | 0.71 | 1.69 | 0.75 | 0.73 | 1.61 | -0.88 | | -0.10 |  |
| SAAFEC-SEQ | 0.51 | 0.80 | 1.36 | -0.32 | 0.18 | 3.19 | 0.19 | 0.49 | 2.45 | 0.73 | | -1.29 |  |
| Fold0 | 0.72 | 0.85 | 1.11 | 0.72 | 0.81 | 1.10 | 0.85 | 0.83 | 1.11 | -0.99 | | 0.02 |  |
| Fold1 | 0.65 | 0.83 | 1.19 | 0.65 | 0.78 | 1.19 | 0.82 | 0.81 | 1.19 | -0.99 | | 0.00 |  |
| Fold2 | 0.49 | 0.80 | 1.44 | 0.50 | 0.78 | 1.43 | 0.72 | 0.79 | 1.43 | -0.97 | | 0.00 |  |
| Fold3 | 0.71 | 0.84 | 1.10 | 0.70 | 0.82 | 1.12 | 0.84 | 0.83 | 1.11 | -0.98 | | 0.01 |  |
| Fold4 | 0.74 | 0.86 | 1.09 | 0.76 | 0.84 | 1.04 | 0.87 | 0.85 | 1.07 | -0.99 | | 0.00 |  |
| Fold_Mean | 0.66 | 0.84 | 1.19 | 0.67 | 0.81 | 1.18 | 0.82 | 0.82 | 1.18 | -0.98 | | 0.01 |  |
| THPLM^E^ | 0.72 | 0.86 | 1.10 | 0.72 | 0.84 | 1.09 | 0.85 | 0.84 | 1.09 | -1.00 | | 0.00 |  |
| THPLM | 0.76 | 0.84 | 1.24 | 0.76 | 0.82 | 1.25 | 0.86 | 0.83 | 1.25 | -1.00 | | -0.02 |  |

Fold_Mean: The averaging performance of Fold0, Fold1, Fold2, Fold3, and Fold4 performances. THPLM^E^ is the ensemble model of Fold0, Fold1, Fold2, Fold3, and Fold4 models.

**Supplementary Table 6**. Atomic-level interactions of 1osj and 1osi on position 172 by RING3.0

| PDBID | Chain | Residue | Interactions | | | | | |
| --- | --- | --- | --- | --- | --- | --- | --- | --- |
|  |  |  | H-bond | Ionic | π-cation | π-π stacking | Disulphide | Van Der Waals |
| 1osi | A | ALA-172 | 3 | 0 | 0 | 0 | 0 | 0 |
| 1osj | A | LEU-172 | 4 | 0 | 0 | 0 | 0 | 2 |

**Supplementary Table 7.** The performances of S^sym^ dataset predicted by five-fold cross-validation models separably, their averaging performance, their averaging predicted DDG performance, Model1426, and THPLM

|  | Direct + reverse | | | Direct | | | Reverse | | | Bias/Antisymmetry | |
| --- | --- | --- | --- | --- | --- | --- | --- | --- | --- | --- | --- |
|  | PCC | ACC | RMSE | PCC | ACC | RMSE | PCC | ACC | RMSE | PCC | <$\delta$> |
| Fold0 | 0.80 | 0.81 | 1.12 | 0.69 | 0.82 | 1.12 | 0.69 | 0.81 | 1.12 | -0.99 | 0.03 |
| Fold1 | 0.77 | 0.78 | 1.18 | 0.65 | 0.79 | 1.18 | 0.66 | 0.78 | 1.18 | -0.99 | -0.01 |
| Fold2 | 0.77 | 0.81 | 1.17 | 0.67 | 0.82 | 1.18 | 0.68 | 0.80 | 1.17 | -0.98 | 0.01 |
| Fold3 | 0.82 | 0.84 | 1.05 | 0.73 | 0.85 | 1.06 | 0.74 | 0.83 | 1.04 | -0.98 | 0.01 |
| Fold4 | 0.86 | 0.86 | 0.95 | 0.79 | 0.87 | 0.97 | 0.80 | 0.86 | 0.94 | -0.99 | 0.00 |
| Fold_Mean | 0.81 | 0.82 | 1.09 | 0.71 | 0.83 | 1.10 | 0.71 | 0.82 | 1.09 | -0.99 | 0.01 |
| THPLM^E^ | 0.85 | 0.84 | 0.99 | 0.77 | 0.85 | 0.99 | 0.78 | 0.82 | 0.98 | -1.00 | 0.01 |
| Model1426 | 0.66 | 0.72 | 1.42 | 0.45 | 0.73 | 1.43 | 0.47 | 0.72 | 1.42 | -0.99 | -0.01 |
| THPLM | 0.78 | 0.82 | 1.24 | 0.68 | 0.81 | 1.23 | 0.66 | 0.82 | 1.25 | -0.98 | -0.02 |

Fold_Mean: The averaging performance of Fold0, Fold1, Fold2, Fold3, and Fold4 performances. THPLM^E^ is the ensemble model of Fold0, Fold1, Fold2, Fold3, and Fold4 models. Model1426 was trained by the rest variations after removing over 25% sequence identity with S^sym^.

**Supplementary Table 8**. Performance of separate protein from Datasets S^sym^ and S^sym^148 between the predicted $\Delta\Delta Gs$ by Model1426 and experimental $\Delta\Delta Gs$

|  |  | Direct + reverse | | | Direct | | | Reverse | | | Bias/Antisymmetry | |
| --- | --- | --- | --- | --- | --- | --- | --- | --- | --- | --- | --- | --- |
| Dataset | PDBID | PCC | ACC | RMSE | PCC | ACC | RMSE | PCC | ACC | RMSE | $r_{d-r}$ | $<\delta>$ |
| 1IHB | 0.85 | 1.00 | 0.43 | 0.94 | 1.00 | 0.46 | 0.94 | 1.00 | 0.41 | -1.00 | -0.04 |  |
| S^sym^ | 1VQB | 0.32 | 0.60 | 1.79 | 0.01 | 0.60 | 1.78 | 0.01 | 0.60 | 1.80 | -1.00 | -0.01 |
|  | 1BNI | 0.74 | 0.85 | 2.12 | 0.27 | 0.85 | 2.14 | 0.30 | 0.85 | 2.10 | -0.99 | 0.02 |
|  | 1L63 | 0.80 | 0.80 | 1.24 | 0.57 | 0.79 | 1.24 | 0.58 | 0.81 | 1.24 | -0.99 | -0.01 |
|  | 2LZM | 0.70 | 0.70 | 1.42 | 0.41 | 0.67 | 1.42 | 0.42 | 0.74 | 1.42 | -0.98 | 0.00 |
|  | 5PTI | 0.88 | 1.00 | 2.76 | -0.19 | 1.00 | 2.79 | -0.04 | 1.00 | 2.74 | -0.99 | -0.07 |
|  | 1CEY | -0.98 | 0.00 | 3.62 | -0.89 | 0.00 | 3.64 | -0.99 | 0.00 | 3.60 | -0.95 | -0.02 |
|  | 1LZ1 | 0.70 | 0.79 | 1.03 | 0.45 | 0.80 | 1.02 | 0.44 | 0.77 | 1.03 | -0.99 | 0.00 |
|  | 1EY0 | 0.04 | 0.39 | 1.78 | -0.15 | 0.36 | 1.79 | -0.09 | 0.43 | 1.77 | -0.99 | -0.01 |
|  | 2RN2 | -0.02 | 0.36 | 1.38 | 0.52 | 0.33 | 1.39 | 0.51 | 0.39 | 1.37 | -0.99 | -0.02 |
|  | 1AMQ | 0.78 | 1.00 | 2.36 | -0.74 | 1.00 | 2.36 | -0.69 | 1.00 | 2.35 | -1.00 | 0.00 |
|  | 1OH0 | -0.31 | 0.50 | 1.10 | 1.00 | 0.50 | 1.09 | 1.00 | 0.50 | 1.10 | -1.00 | 0.01 |
|  | 4LYZ | 0.47 | 0.82 | 0.87 | 0.26 | 0.86 | 0.87 | 0.27 | 0.79 | 0.88 | -1.00 | -0.01 |
| S^sym^148 | 2LZM | 0.88 | 0.88 | 1.82 | 0.64 | 0.82 | 1.82 | 0.68 | 0.94 | 1.81 | -0.98 | 0.00 |
|  | 1L63 | 0.80 | 0.80 | 1.24 | 0.57 | 0.79 | 1.24 | 0.58 | 0.81 | 1.24 | -0.99 | -0.01 |
|  | 5PTI | 0.95 | 1.00 | 2.79 | -0.08 | 1.00 | 2.77 | -0.02 | 1.00 | 2.81 | -1.00 | -0.03 |
|  | 1LZ1 | 0.65 | 0.60 | 0.80 | 0.57 | 0.60 | 0.80 | 0.63 | 0.60 | 0.80 | -0.97 | 0.04 |
|  | 1EY0 | -0.63 | 0.17 | 1.06 | 0.64 | 0.00 | 1.08 | 0.64 | 0.33 | 1.04 | -1.00 | -0.02 |

**Supplementary Table 9**. Hypermeters for THPLM

| layer | Hypermeters | Value |
| --- | --- | --- |
| ESM-2 | layers | 36 |
|  | Model name | esm2_t36_3B_UR50D |
|  | Batch size | Based on ESM-2 |
| CNN-1 | Channel | 8 |
|  | Padding | 1 |
|  | Kernel size | 3×1 |
|  | stride | 1 |
|  | Activate function | ReLU |
|  | Batch Normalization | 8 |
| CNN-2 | Channel | 32 |
|  | Padding | 1 |
|  | Kernel size | 3×1 |
|  | stride | 1 |
|  | Activate function | ReLU |
|  | Batch Normalization | 32 |
| Linear-1 | Input dimension | 32×2560 |
|  | Output dimension | 128 |
|  | Activate function | ReLU |
|  | Batch Normalization | 128 |
| Linear-1 | Input dimension | 128 |
|  | Output dimension | 1 |

**Supplementary Table 10**. Variation count of each protein from Datasets S^sym^ and S^sym^148

| Dataset | PDBID | variation count |
| --- | --- | --- |
| Ssym | 1L63 | 118 |
|  | 2LZM | 66 |
|  | 1LZ1 | 61 |
|  | 1EY0 | 28 |
|  | 2RN2 | 18 |
|  | 4LYZ | 14 |
|  | 1BNI | 13 |
|  | 1VQB | 5 |
|  | 5PTI | 5 |
|  | 1AMQ | 4 |
|  | 1CEY | 3 |
|  | 1IHB | 3 |
|  | 1OH0 | 2 |
|  | 1IOB | 1 |
|  | 1RN1 | 1 |

**Supplementary Table 11**. Variation count of each protein from Datasets S^sym^ and S^sym^148

| Dataset | PDBID | variation count |
| --- | --- | --- |
| Ssym148 | 1L63 | 118 |
|  | 2LZM | 17 |
|  | 1LZ1 | 5 |
|  | 1EY0 | 3 |
|  | 5PTI | 3 |
|  | 1IOB | 1 |
|  | 2RN2 | 1 |

**Supplementary Table 12.** The performances of models with different embeddings from different parameters of ESM-2

|  | Direct + reverse | | | Direct | | | Reverse | | | | Bias/Antisymmetry | | |
| --- | --- | --- | --- | --- | --- | --- | --- | --- | --- | --- | --- | --- | --- |
| Model | PCC | ACC | RMSE | PCC | ACC | RMSE | PCC | ACC | RMSE | $r_{d-r}$ | | $<\delta>$ |  |
| t6_8M | 0.68 | 0.77 | 1.28 | 0.57 | 0.76 | 1.3 | 0.58 | 0.77 | 1.27 | -0.92 | | 0.03 |  |
| t12_35M | 0.74 | 0.81 | 1.15 | 0.63 | 0.81 | 1.15 | 0.62 | 0.81 | 1.15 | -0.95 | | 0.02 |  |
| t30_150M | 0.78 | 0.83 | 1.06 | 0.68 | 0.83 | 1.07 | 0.69 | 0.84 | 1.04 | -0.95 | | 0.00 |  |
| t33_650M | 0.85 | 0.85 | 0.9 | 0.77 | 0.85 | 0.9 | 0.77 | 0.85 | 0.9 | -1.00 | | 0.00 |  |
| t36_3B | 0.86 | 0.87 | 0.87 | 0.78 | 0.87 | 0.87 | 0.79 | 0.87 | 0.87 | -1.00 | | 0.03 |  |

Embeddings from pretrained model ESM-2 with different parameters are esm2_t36_3B_UR50D (t36_3B), esm2_t33_650M_UR50D(t33_650M), esm2_t30_150M_UR50D(t30_150M), esm2_t12_35M_UR50D(t12_35M), and esm2_t6_8M_UR50D(t6_8M). Then we used embeddings to train new models t36_3B, t33_650M, t30_150M, t12_35M, and t6_8M separately using the MLPRegressor algorithm from scikit-learn to predict stability changes.

**Supplementary Table 13**. Performance of THPLM across four testing datasets

|  | Direct | | | Reverse | | | Direct + reverse | | | Bias/Antisymmetry | |
| --- | --- | --- | --- | --- | --- | --- | --- | --- | --- | --- | --- |
|  | PCC | ACC | RMSE | PCC | ACC | RMSE | PCC | ACC | RMSE | PCC | <$\delta$> |
| S350 | 0.72 | 0.81 | 1.11 | 0.72 | 0.82 | 1.1 | 0.79 | 0.82 | 1.1 | -0.99 | 0.03 |
| S^sym^148 | 0.76 | 0.84 | 1.24 | 0.76 | 0.82 | 1.25 | 0.86 | 0.83 | 1.25 | -1.00 | -0.02 |
| S669 | 0.39 | 0.74 | 1.60 | 0.35 | 0.73 | 1.66 | 0.53 | 0.74 | 1.63 | -0.96 | -0.01 |
| Frataxin | 0.67 | 0.75 | 3.55 | 0.72 | 0.63 | 3.55 | 0.80 | 0.69 | 3.55 | -1.00 | -0.02 |

**Supplementary Table 14**. Parameters for running RING3.0 to get atomic-level interactions of 1osj and 1osi

| Input file | Chain | Nodes | Edges | Distance thresholds(Å) | | | | | |
| --- | --- | --- | --- | --- | --- | --- | --- | --- | --- |
|  |  |  |  | H-bond | Ionic | π-cation | π-π stacking | Disulphide | Van Der Waals |
| 1osi.pdb | A | closest | All | 3.5 | 4 | 5 | 6.5 | 2.5 | 0.5 |
| 1osj.pdb | A | closest | All | 3.5 | 4 | 5 | 6.5 | 2.5 | 0.5 |

# Supplementary Figures


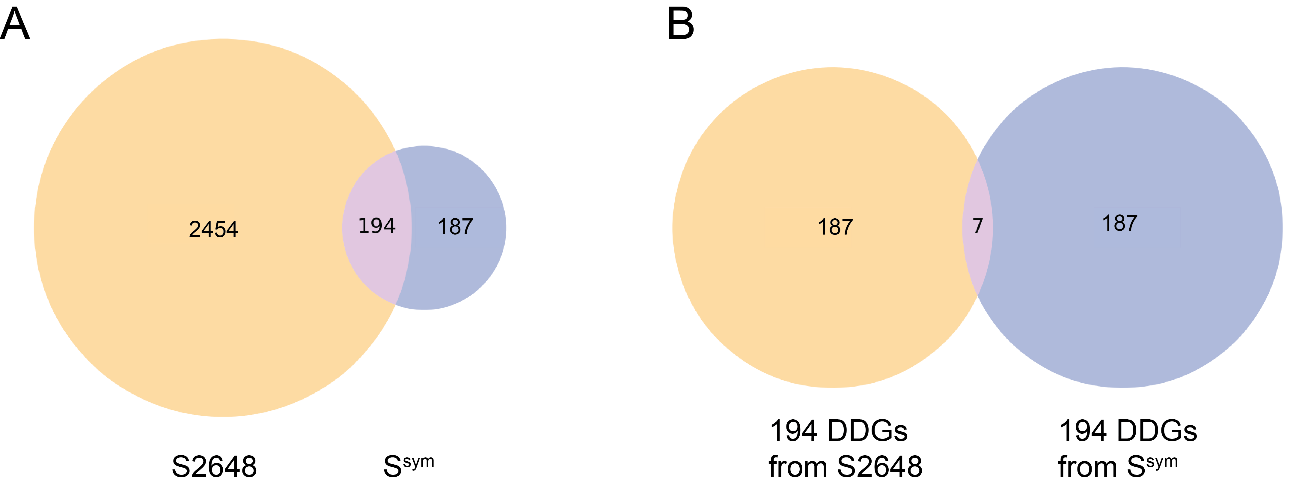


**Supplementary Figure 1.** Analysis for the overlapped variations between S2648 and S^sym^. **(A)** Overlapped variations between S2648 and S^sym^. **(B)** The overlapped ΔΔG (DDG) between 194 overlapped variations.


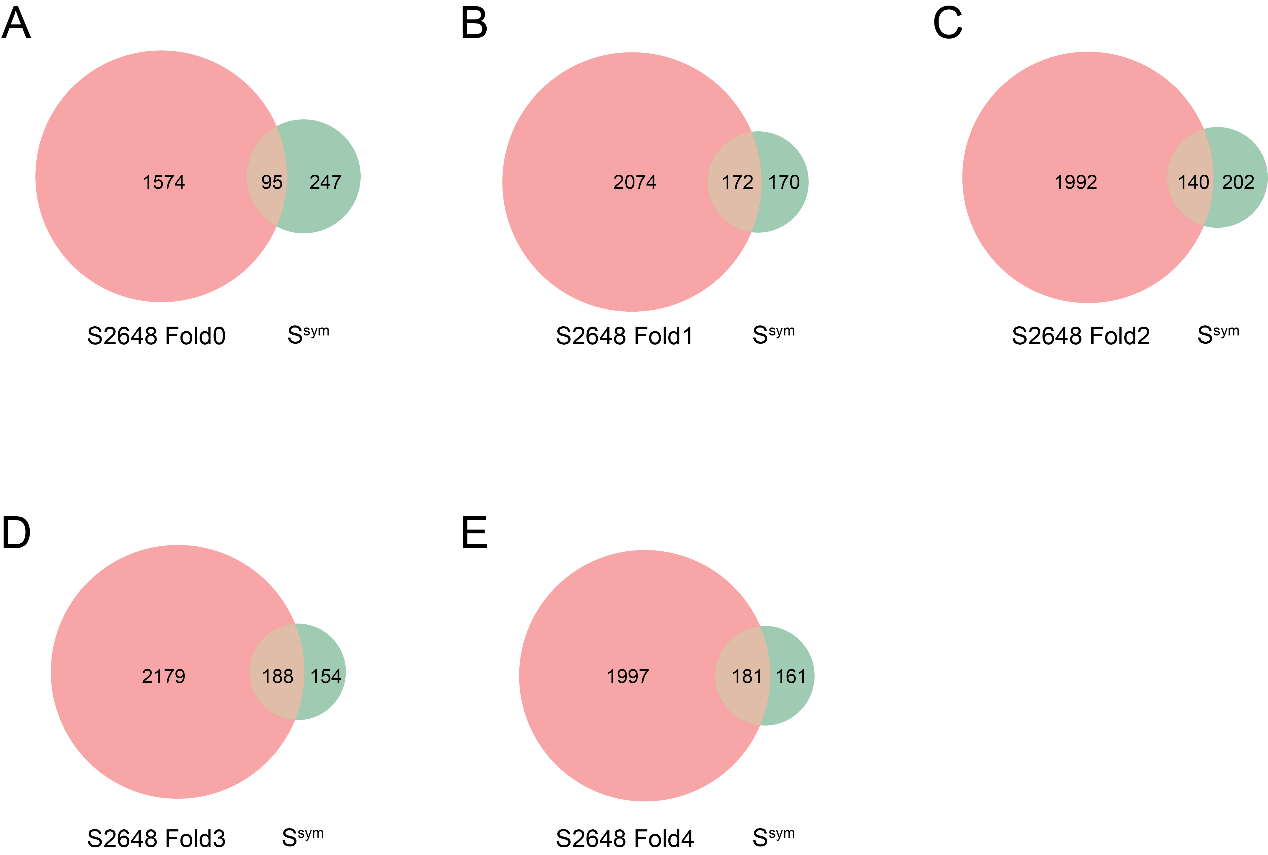


**Supplementary Figure 2.** The overlaps between the S^sym^ and five training datasets from S2648 by authors (Fariselli, et al., 2015)


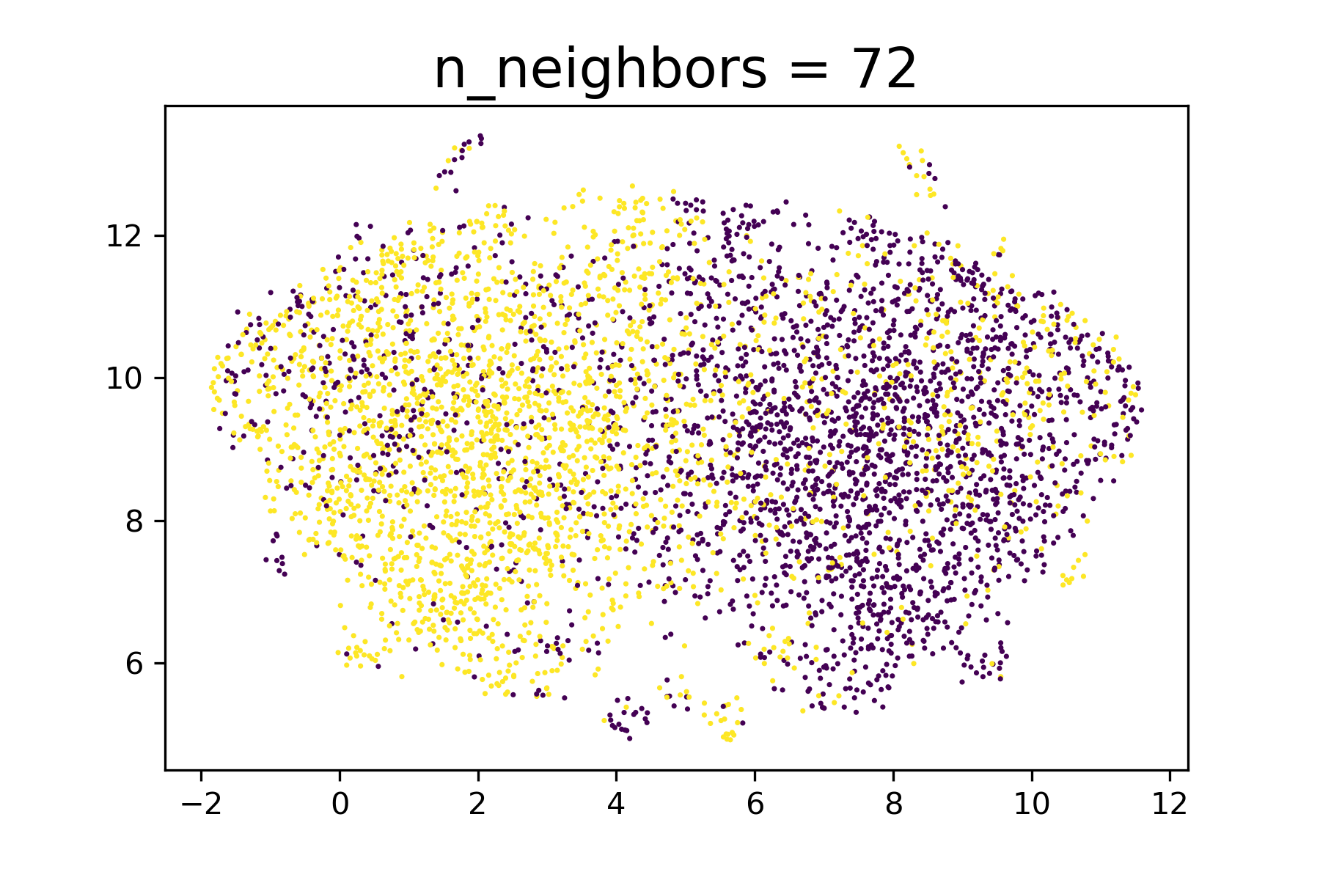


**Supplementary Figure 3.** UMAP plot of features, subtraction between variant and wild-type sequences, with ΔΔG as labels (Yellow points for negative variations, and purple points for positive variations).
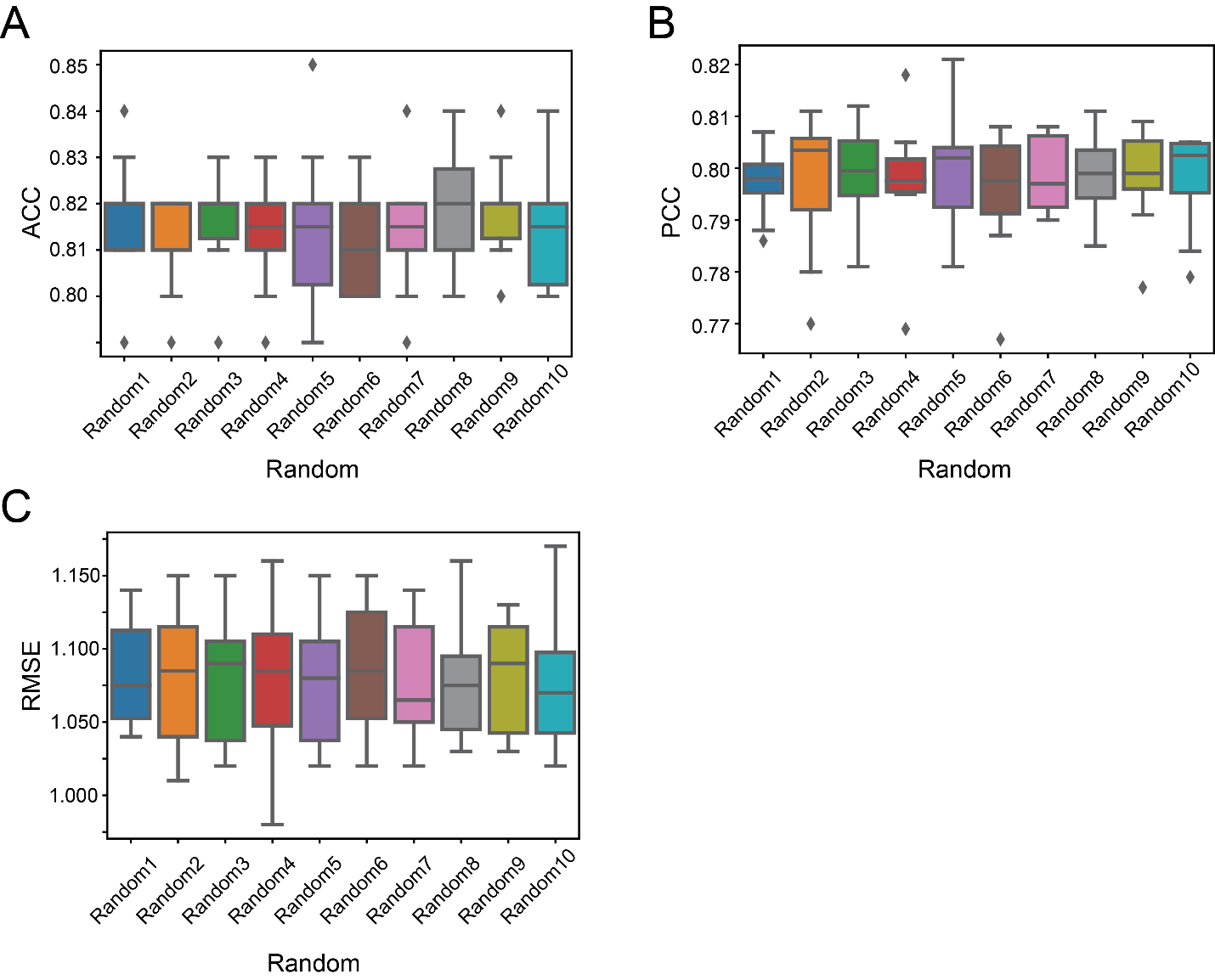


**Supplementary Figure 4.** The boxplot of ACC、PCC and RMSE on 10 times 10-fold cross-validation of THPLM.

# References

Benevenuta, S.*, et al.* An antisymmetric neural network to predict free energy changes in protein variants. *J Phys D Appl Phys* 2021;54(24).

Capriotti, E., Fariselli, P. and Casadio, R. I-Mutant2.0: predicting stability changes upon mutation from the protein sequence or structure. *Nucleic Acids Res* 2005;33(Web Server issue):W306-310.

Chen, Y.*, et al.* PremPS: Predicting the impact of missense mutations on protein stability. *PLoS Comput Biol* 2020;16(12):e1008543.

Cheng, J., Randall, A. and Baldi, P. Prediction of protein stability changes for single-site mutations using support vector machines. *Proteins* 2006;62(4):1125-1132.

Fariselli, P.*, et al.* INPS: predicting the impact of non-synonymous variations on protein stability from sequence. *Bioinformatics* 2015;31(17):2816-2821.

Folkman, L.*, et al.* EASE-MM: Sequence-Based Prediction of Mutation-Induced Stability Changes with Feature-Based Multiple Models. *J Mol Biol* 2016;428(6):1394-1405.

Gong, J.*, et al.* Prediction of protein stability changes upon single-point variant using 3D structure profile. *Comput Struct Biotec* 2023;21:354-364.

Guerois, R., Nielsen, J.E. and Serrano, L. Predicting changes in the stability of proteins and protein complexes: a study of more than 1000 mutations. *Journal of molecular biology* 2002;320(2):369-387.

Kellogg, E.H., Leaver‐Fay, A. and Baker, D. Role of conformational sampling in computing mutation‐induced changes in protein structure and stability. *Proteins: Structure, Function, and Bioinformatics* 2011;79(3):830-838.

Kingma, D.P. and Ba, J. Adam: A method for stochastic optimization. *arXiv preprint arXiv:1412.6980* 2014.

Laimer, J.*, et al.* MAESTRO--multi agent stability prediction upon point mutations. *BMC Bioinformatics* 2015;16:116.

Li, G., Panday, S.K. and Alexov, E. SAAFEC-SEQ: A Sequence-Based Method for Predicting the Effect of Single Point Mutations on Protein Thermodynamic Stability. *Int J Mol Sci* 2021;22(2).

Montanucci, L.*, et al.* DDGun: an untrained method for the prediction of protein stability changes upon single and multiple point variations. *BMC Bioinformatics* 2019;20(Suppl 14):335.

Pancotti, C.*, et al.* Predicting protein stability changes upon single-point mutation: a thorough comparison of the available tools on a new dataset. *Briefings in Bioinformatics* 2022;23(2).

Pancotti, C.*, et al.* A Deep-Learning Sequence-Based Method to Predict Protein Stability Changes Upon Genetic Variations. *Genes-Basel* 2021;12(6).

Pandurangan, A.P.*, et al.* SDM: a server for predicting effects of mutations on protein stability. *Nucleic Acids Res* 2017;45(W1):W229-W235.

Pires, D.E., Ascher, D.B. and Blundell, T.L. DUET: a server for predicting effects of mutations on protein stability using an integrated computational approach. *Nucleic Acids Res* 2014;42(Web Server issue):W314-319.

Pires, D.E., Ascher, D.B. and Blundell, T.L. mCSM: predicting the effects of mutations in proteins using graph-based signatures. *Bioinformatics* 2014;30(3):335-342.

Pucci, F.*, et al.* Quantification of biases in predictions of protein stability changes upon mutations. *Bioinformatics* 2018;34(21):3659-3665.

Rodrigues, C.H., Pires, D.E. and Ascher, D.B. DynaMut: predicting the impact of mutations on protein conformation, flexibility and stability. *Nucleic Acids Res* 2018;46(W1):W350-W355.

Rodrigues, C.H.M., Pires, D.E.V. and Ascher, D.B. DynaMut2: Assessing changes in stability and flexibility upon single and multiple point missense mutations. *Protein Sci* 2021;30(1):60-69.

Savojardo, C.*, et al.* INPS-MD: a web server to predict stability of protein variants from sequence and structure. *Bioinformatics* 2016;32(16):2542-2544.

Worth, C.L., Preissner, R. and Blundell, T.L. SDM--a server for predicting effects of mutations on protein stability and malfunction. *Nucleic Acids Res* 2011;39(Web Server issue):W215-222.
